# *BRAF* Mutations in Patients with Myeloid Neoplasms: A Cancer Center Multigene Next-Generation Sequencing Analysis Experience

**DOI:** 10.3390/ijms25105183

**Published:** 2024-05-09

**Authors:** Fei Fei, Caitlin Caporale, Lisa Chang, Barbara K. Fortini, Haris Ali, Diana Bell, Anthony Stein, Guido Marcucci, Milhan Telatar, Michelle Afkhami

**Affiliations:** 1 Department of Pathology, City of Hope Comprehensive Cancer Center, Duarte, CA 91010, USA; ffei@coh.org (F.F.);; 2 Breast Oncology, Dana-Farber Cancer Institute, Boston, MA 02215, USA; caitlin_caporale@dfci.harvard.edu; 3 Keck Graduate Institute, Claremont, CA 91711, USA; 4 Department of Hematology & Hematopoietic Cell Transplantation, City of Hope Comprehensive Cancer Center, Duarte, CA 91010, USA; 5 Department of Pathology, University of Pittsburgh Medical Center, Pittsburgh, PA 15213, USA

**Keywords:** *BRAF* mutation, myeloid neoplasms, next-generation sequencing

## Abstract

*BRAF* mutations are rare in myeloid neoplasms and are reported to be associated with poor treatment outcomes. The purpose of our study is to characterize *BRAF* mutations in myeloid neoplasms using a next-generation sequencing (NGS) panel based on the experiences of a single cancer center. We conducted a retrospective review of patients with myeloid neoplasms who underwent the HopeSeq studies between January 2018 and September 2023. A total of 14 patients with myeloid neoplasms carrying *BRAF* mutations were included in our cohort. The clinical, pathological, and molecular features of these patients were investigated. Our study indicates that *BRAF* mutations are rare in myeloid neoplasms, constituting only 0.53% (14/2632) of all myeloid neoplasm cases, with the most common *BRAF* mutation being *BRAF* V600E (4/14; 28.6%). Interestingly, we observed that six out of seven patients with acute myeloid leukemia (AML) exhibited AML with monocytic differentiation, and all the patients with AML exhibited an extremely poor prognosis compared to those without *BRAF* mutations. *TET2* (5/14; 35.7%), *ASXL1* (4/14; 28.6%), and *JAK2* (4/14; 28.6%) were the three most frequently co-mutated genes in these patients. Moreover, we noted concurrent *KMT2A* gene rearrangement with *BRAF* mutations in three patients with AML (3/7; 42.9%). Our study suggests that although *BRAF* mutations are rare in myeloid neoplasms, they play a crucial role in the pathogenesis of specific AML subtypes. Furthermore, RAS pathway alterations, including *BRAF* mutations, are associated with *KMT2A* gene rearrangement in AML. However, these findings warrant further validation in larger studies.

## 1. Introduction

*BRAF* is an oncogene and a member of the Raf family of serine/threonine protein kinases, playing a pivotal role in regulating the mitogen-activated protein kinase (MAPK) pathway, which, in turn, influences both cell proliferation and survival [1]. *BRAF* is one of the most mutated kinases in human cancers, particularly in melanoma, with a mutation rate of 40–50%. In addition, *BRAF* mutations are frequently observed in thyroid cancer, as well as in a small fraction of lung and colorectal cancers [1]. In hematologic malignancies, *BRAF* mutations have been frequently identified in hairy cell leukemia (HCL), Erdheim–Chester disease, Langerhans cell histiocytosis, and plasma cell neoplasms [2]. However, the significance of *BRAF* mutations in myeloid neoplasms has not been widely investigated [3,4,5,6].

The clinically significant *BRAF* missense mutations are predominantly located in the tyrosine kinase domain, between exon 11 and exon 15, particularly within the glycine-rich loop and activation segment, respectively. Among these mutations, the *BRAF* V600E mutation accounts for 80% of *BRAF* mutations identified in human cancers [7]. A recent study by Ping et al. indicated the absence of *BRAF* mutations involving exon 15 in acute myeloid leukemia (AML), myelodysplastic syndromes (MDS), and myeloproliferative neoplasms (MPN) within a cohort of 578 patients with myeloid neoplasms [4]. Furthermore, a meta-analysis of gene mutation profiles in MDS, MDS/MPN, and MPN revealed that the frequency of *BRAF* mutations is less than 1% in these cases [8]. Despite the rarity of *BRAF* mutations in myeloid neoplasms, Kamata et al.’s study suggests that *BRAF* plays a critical role in myeloid progenitor cell formation and megakaryocytopoiesis [9]. Additionally, Christiansen et al. found that *BRAF* mutations are important in the pathogenesis of specific AML subtypes [5]. Zhang et al. indicated that a subset of patients with RAS wild-type chronic myelomonocytic leukemia (CMML) harbors BRAF kinase domain mutations that are potentially capable of activating the MAPK signaling pathway [10]. These findings indicate a potential association of *BRAF* mutations with myeloid neoplasms.

The purpose of this study is to characterize the *BRAF* mutations in myeloid neoplasms using a multigene next-generation sequencing (NGS) assay based on the experiences of a single cancer center.

## 2. Results

### 2.1. Case cohort Characteristics

A total of 2632 patients with myeloid neoplasms were identified between January 2018 and September 2023. Pathogenic or likely pathogenic *BRAF* mutations were identified in 14 patients, resulting in a prevalence of 0.53% (14/2632) in myeloid neoplasms. The possibility of HCL was ruled out through additional flow cytometry studies or immunostaining for CD11c, CD103, CD25, etc. The mean age of the patients was 63.9 years (range: 23–89 years), with ten males and four females. The most common diagnoses were AML (7/14; 50%), followed by MPN (4/14; 28.6%) and MDS (3/14; 21.4%). Cytogenetic abnormalities were identified in 10 out of 11 cases (90.9%). Furthermore, six out of seven patients with AML exhibited AML with monocytic differentiation, and a complex karyotype was observed in five out of six patients with AML with available cytogenetic studies. The clinical and pathological features of the patients are summarized in Table 1.

### 2.2. Mutation Profiles of Patients with BRAF Mutations

**Figure 1:**
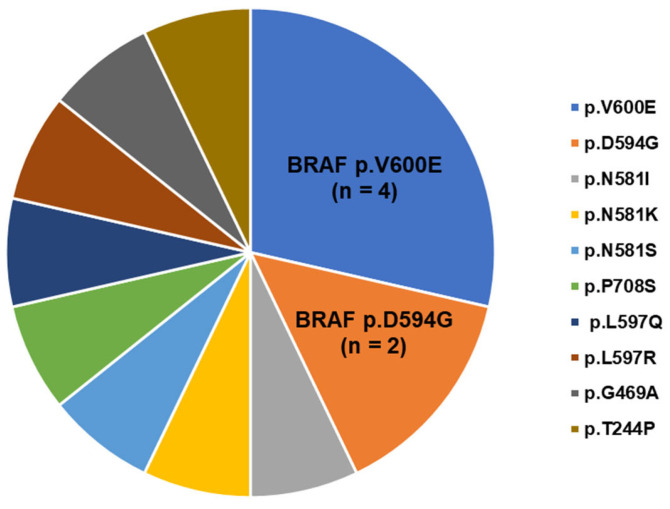
*BRAF* mutations in patients with myeloid neoplasms (n = 14).

As illustrated in Figure 1, the most common *BRAF* mutations were *BRAF* V600E (4/14; 28.6%), followed by D594G (2/14; 14.3%), N581S (1/14; 7.1%), N581I (1/14; 7.1%), and N581K (1/14; 7.1%). The average variant allele frequency (VAF) was 31.8%, ranging from 3% to 90%. This suggests that *BRAF* mutations were found both as dominant clonal and subclonal events. The majority of *BRAF* mutations (12/14; 85.7%) were located in exon 15, within the kinase domain of the protein.

**Figure 2:**
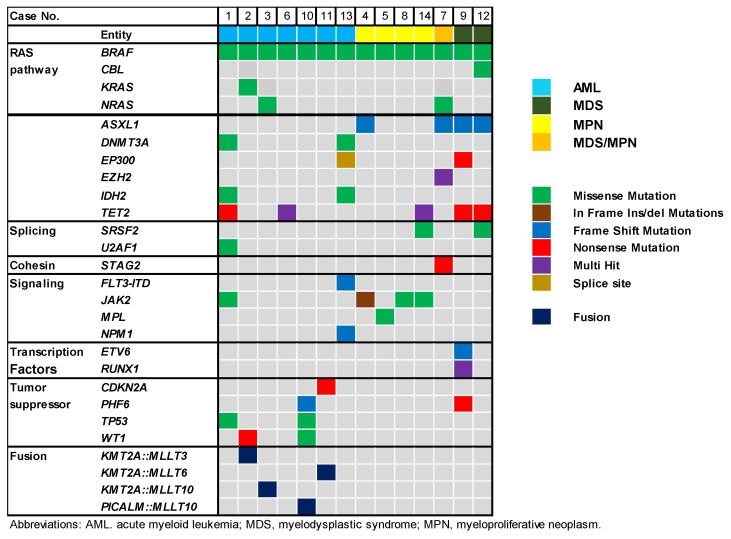
Pathogenic/likely pathogenic mutations identified in myeloid neoplasms with *BRAF* mutations (n = 14).

In the next step, we investigated the mutation profiles of the patients with *BRAF* mutations. The gene mutation profiles of these 14 patients are summarized in Table 1/Figure 2. *TET2* (5/14; 35.7%), *ASXL1* (4/14; 28.6%), and *JAK2* (4/14; 28.6%) were the three most frequently mutated genes in our cohort. *JAK2* was identified in three out of four patients with MPN and one patient with secondary AML arising from MPN, consisting with the disease characteristics. Interestingly, *FLT3-ITD* was identified in only one patient with AML (1/7; 14.3%), with no *FLT3-TKD* detected in our cohort. Furthermore, we observed that the *BRAF* mutation remained stable or increased in three patients with available relapse specimens (Case Nos. 4, 5, and 11). In one patient, the *BRAF* mutation was lost during relapse, while the rest of RAS-related genes (*KRAS* and *WT1*), and the *KMT2A::MLLT3* fusion persisted (Case No. 2), suggesting that the *BRAF* mutation may not be the driver mutation for this leukemia. Additionally, we observed that concurrent *KMT2A* gene rearrangement with *BRAF* mutations in 3 patients with AML (3/7; 42.9%) (Case Nos. 2, 3, and 11), including *BRAF* L597Q, D594G, and V600E.

### 2.3. KMT2A Gene Rearrangement in AML

**Figure 3:**
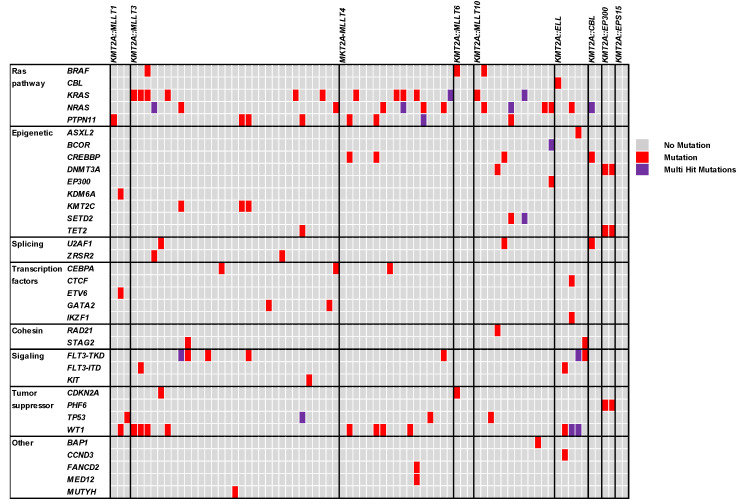
Pathogenic/likely pathogenic mutations identified in AML patients with *KMT2A* gene rearrangements (n = 77).

To further characterize the association between *KMT2A* gene rearrangement and *BRAF* mutations, we conducted a retrospective review of our HopeSeq Heme panel database and identified 77 AML patients with the *KMT2A* gene rearrangement. The gene mutational profiles of these AML patients are summarized in Figure 3. Among the 77 AML patients with *KMT2A* gene rearrangements, the most frequent abnormality was *KMT2A::MLLT3* (31/77; 40.3%), followed by *KMT2A::MLLT4* (17/77; 22.1%), *KMT2A::MLLT10* (12/77; 15.6%), and *KMT2A::ELL* (4/77; 5.2%). Consistent with the previous studies, the most commonly mutated genes were *KRAS* (13/77; 16.9%), *NRAS* (13/77; 16.9%), *WT1* (12/77; 15.6%), *PTPN11* (8/77; 10.4%), and *FLT3-TKD* (7/77; 9.1%) [11]. Overall, mutations in genes constituting the RAS pathway (*KRAS*, *NRAS*, *PTPN11*, and *BRAF*) were identified in 34 patients with the *KMT2A* gene rearrangement (33/77; 42.9%), and most of these alterations were mutually exclusive (28/33; 84.8%) [11].

### 2.4. Survival Analysis of Patients with BRAF Mutations

**Figure 4:**
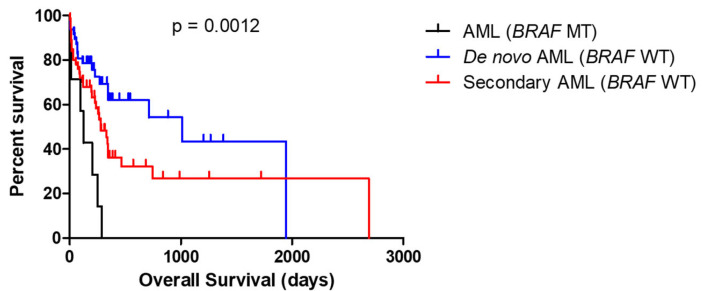
Overall survival between AML patients with or without BRAF mutations.

In the next step, we investigated the clinical outcomes of the AML patients harboring *BRAF* mutations. We randomly selected 50 patients with de novo AML and 50 patients with secondary AML without *BRAF* mutations. As illustrated in Figure 4, the AML patients with *BRAF* mutations showed an extremely poor prognosis (n = 7) compared to that of the de novo AML patients or secondary AML patients without *BRAF* mutations, with a median survival time of 126 days, ranging from 2 to 290 days (*p* = 0.0012). Consistent with the previous studies, the patients with secondary AML show an unfavorable prognosis compared to the patients with de novo AML (Figure 4). However, regardless of whether the patients had de novo AML or secondary AML, AML patients with *BRAF* mutations all showed extremely poor prognosis. Additionally, no significant difference in overall survival was observed among the AML patients with different *BRAF* mutation subtypes. The patients’ clinical outcomes, including treatment responses were summarized in Supplementary Table S3.

## 3. Materials and Methods

### 3.1. Patients and Specimens

This study was approved by the City of Hope Comprehensive Cancer Center Review Board (IRB #15198). We conducted a retrospective review of patients with myeloid neoplasms who underwent different versions of the HopeSeq NGS assay between January 2018 and September 2023 at the CLIA-approved clinical molecular diagnostics laboratory. A total of 2632 patients were identified, and their clinical, pathological, and molecular findings were reviewed by two hematopathologists for this study.

### 3.2. HopeSeq Heme Panel (HopeSeq)

The various versions of the DNA-based HopeSeq Heme panels cover a range of 73 to 523 genes, all including the entire coding exons of the *BRAF* gene. This assay detects single-nucleotide variants (SNVs), insertions/deletions (indels), copy number variants (CNVs), and splice site variants. Peripheral blood, bone marrow aspirates, and bone marrow clot sections were used as inputs for the HopeSeq Heme panels, with a requirement of 40 ng DNA. For consistency of comparison, we analyzed only the 73 genes listed in Supplementary Table S1. These genes are recurrently altered in myeloid and lymphoid neoplasms and were selected based on the literature and clinicians’ requirements.

The workflow includes the acoustic shearing of isolated genomic DNA, library preparation, and the subsequent enrichment of specific genes of interest using a capture-based method. The normalized and enriched libraries were pooled, clustered on the flow cells, and then sequenced on the Illumina NextSeq 550. The Local Run Manager TruSight Oncology Comprehensive analysis module was utilized to analyze the sequencing results.

### 3.3. Statistical Analysis

Baseline characteristics are presented as mean and range for continuous variables and frequency for categorical variables. Overall survival (OS) was defined as time from diagnosis to the last follow-up or death from any cause. Survival curves were calculated using the log-rank test. All the data were analyzed using GraphPad Prism 5 software.

## 4. Discussion

*BRAF* mutations are rare in myeloid neoplasms and are reported to be associated with poor treatment outcomes in patients with AML carrying *BRAF* mutations [12]. However, no comprehensive analysis has been conducted on the clinical and molecular characteristics of *BRAF* mutations in patients with myeloid neoplasms. In this study, we utilized a comprehensive NGS panel to characterize the *BRAF* mutations and co-occurring mutations in myeloid neoplasms. These findings highlight the need for targeted therapies for patients with specific *BRAF* mutations.

In our study, we found that *BRAF* mutations constitute 0.53% of myeloid neoplasms, consistent with previous studies reporting values from approximately 0% to 0.65% [3,4,5]. Abu-Shihab et al.’s study indicates that the most frequent co-occurring mutations in *BRAF*-mutated AML were *TET2* (36%), *ASXL1* (33%), *NRAS* (29%), *KRAS* (26%), and *RUNX1* (19%) [12]. In our study, we found that *TET2* (29%), *DNMT3A* (29%), *IDH2* (29%), *TP53* (29%), and *WT1* (29%) were the most frequent co-occurring mutations with *BRAF* in AML. We attribute this discrepancy to the limited sample size in our study.

Previous studies have indicated that *FLT3-TKD* and *FLT3-ITD* are among the most common mutations detected in hematological malignancies, particularly in AML. The AML patients with *FLT3* mutations are associated with a higher relapse rate and an inferior overall survival [13]. However, in our cohort, *FLT3-ITD* was identified in only one patient with AML (1/7), suggesting a deficiency in the FLT3 pathway in the leukemogenesis of the *BRAF*-mutated patients with AML. Abu-Shihab et al. indicates that *BRAF*-mutated AML is rare and associated with a poor prognosis regardless of the clonal burden and treatment [12]. In line with their findings, we observed that the *BRAF*-mutated AML patients exhibited an extremely poor prognosis compared to those without *BRAF* mutations, regardless of whether they had de novo AML or secondary AML, the specific subtypes of *BRAF* mutation, or the different chemotherapy regimens.

A study by Christiansen et al. suggested a significant association between the RTK/RAS-BRAF pathway and monocytic AML (M5 FAB subtype), as well as a complex karyotype in the patients with AML [5]. Additionally, Xu et al. identified four out of 399 AML patients with a *BRAF* mutation, all of whom had de novo AML with monocytic differentiation [14]. Consistent with these findings, we observed that six out of seven AML cases exhibited monocytic differentiation, and a complex karyotype was observed in five out of six AML cases. Moreover, Christiansen et al. demonstrated a highly significant association between the *BRAF* V600E mutation and *KMT2A* gene rearrangement in therapy-related AML based on three cases [5]. However, no *KMT2A* gene rearrangement was identified in any of three de novo AML cases with *BRAF* mutation in Xu et al.’s study [14]. Interestingly, all three of our *KMT2A* gene rearrangement AML cases with *BRAF* mutation were de novo AML. This discrepancy could be partly attributed to the relatively rare occurrence of *BRAF* mutation in patients with AML. These findings suggest the importance of *BRAF* mutations in the pathogenesis of a specific AML phenotype.

*KMT2A*, also known as lysine methyltransferase 2A, is a transcriptional coactivator that epigenetically regulates gene transcription via methylation and is primarily associated with hematopoietic and embryonic development [15]. The *KMT2A* gene rearrangement occurs in approximately 3–7% of adult patients with de novo AML [11,16]. Bill et al. investigated the mutational status in 96 de novo AML patients with *KMT2A* gene rearrangement and found that 32% of patients had mutations in genes constituting the RAS signaling pathway (*NRAS*, *KRAS*, and *PTPN11*) [11]. Additionally, Lavallée et al.’s study confirmed that 45% of AML patients with *KMT2A* gene rearrangement were mutated for components of the RAS pathway, with a *BRAF* mutation rate of 3.2% (1/31) [17]. This finding is similar to our observation of a *BRAF* mutation rate of 3.9% (3/77) in AML patients with *KMT2A* gene rearrangement.

Our study has some limitations. Firstly, our cohort only includes 14 cases with *BRAF* mutations; thus, large prospective studies are needed to further validate our findings. Secondly, our institution is a tertiary cancer center, and there may be a selection bias towards more aggressive diseases. Thirdly, not all these cases were tested at the initial time of disease. Thus, even with a review of the VAF, we cannot conclusively determine whether *BRAF* is a founder mutation in these cases or a passenger mutation that emerges later in the course of the disease [18].

## 5. Conclusions

Thus, our study indicates that although *BRAF* mutations are rare in myeloid neoplasms, constituting only 0.53% of cases in our cohort, they play a crucial role in the pathogenesis of specific AML subtypes. Furthermore, we found that RAS pathway alterations, including *BRAF* mutations, are associated with *KMT2A* gene rearrangement in AML. Moreover, we observed that AML patients with *BRAF* mutations exhibited an extremely poor prognosis compared to those without *BRAF* mutations, regardless of whether they had de novo AML or secondary AML, the specific subtypes of *BRAF* mutation, or the different chemotherapy regimens. These findings highlight the need to investigate *BRAF* or RAS pathway inhibitors for patients with myeloid neoplasms harboring *BRAF* mutations, particularly for patients with AML carrying *BRAF* mutations. However, our findings need to be validated in large prospective studies.

## Figures and Tables

**Table 1:** Clinical and molecular findings in patients with myeloid neoplasms carrying *BRAF* mutations (n = 14).

Case No.	Age	Sex	Specimen	Diagnosis				Co-mutations (Allele Frequency %)	Fusion
					Genomic Alterations	Allele Frequency	Exon		
1	75	F	BM	Secondary AML arising from MPN	c.2122C>T; p.P708S	78%	17	*JAK2* p.V617F (86%); *TET2* p.Q652* (76%) *TP53* p.R273C (61%); *U2AF1* p.Q157R (44%) *DNMT3A* p.R882H (42%); *IDH2* p.R140Q (9%)	Negative
2	60	M	BM	Acute monocytic leukemia	c.1790T>A; p.L597Q	10%	15	*KRAS* p.G12S (VAF: 22%) *WT1* p.381* (31%)	*KMT2A::MLLT3*
3	45	M	PB	AML with monocytic differentiation	c.1781A>G; p.D594G	29%	15	*NRAS* p.Q61R (35%)	*KMT2A::MLLT10*
4	56	M	PB	MPN	c.1742A>G; p.N581S	43%	15	*ASXL1* p.D855Afs*11 (41%) *JAK2* p.R541_E543delinsK (36%)	Negative
5	80	F	BM	MPN	c.1742A>T; p.N581I	43%	15	*MPL* p.W515L (90%)	Negative
6	83	M	BM	AML with monocytic differentiation	c.1799T>A; p.V600E	27%	15	*TET2* p.R1465* (46%) *TET2* p.S293fs*14 (42%)	Negative
7	89	M	BM	MDS/MPN	c.1743T>A; p.N581K	38%	15	*ASXL1* p.E635Rfs*15 (26%); *EZH2* p.R690H (45%) *EZH2* p.R583* (48%); *NRAS* p.G13D (46%) *STAG2* p.R110* (84%)	Negative
8	75	F	PB	ET	c.1781A>G; p.D594G	16%	15	*JAK2* p.V617F (9%)	Negative
9	77	M	BM	MDS	c.1799T>A; p.V600E	11%	15	*ASXL1* p.T769Pfs*3 (37%); *EP300* p.W1681* (12%) *ETV6* p.E154Gfs*3 (19%); *PHF6* p.S2* (64%) *RUNX1* p.P304Ffs*297 (14%); *RUNX1* p.S389Rfs*213 (18%) *TET2* p.K1299* (14%)	Negative
10	23	M	BM	AML	c.1406G>C; p.G469A	8%	11	*PHF6* p.N23Afs*14 (90%) *TP53* p.R248Q (87%) *WT1* p.R462W (43%)	*PICALM::MLLT10*
11	36	M	BM	AML with monocytic differentiation	c.1799T>A; p.V600E	58%	15	*CDKN2A* p.R80* (60%)	*KMT2A::MLLT6*
12	78	M	PB	MDS	c.1790T>G; p.L597R	8%	15	*ASXL1* p.G646fs*12 (27%); *CBL* p.R420Q (3%) *SRSF2* p.P95L (47%); *TET2* p.Q644* (90%)	Negative
13	45	F	PB	AML	c.1799T>A; p.V600E	15%	15	*DNMT3A* p.R882H (36%); *EP300* c.3671+1G>A (16%) *IDH2* p.R140Q (3%) *FLT3-ITD (N/A); NPM1* p.W288Cfs*12 (<1%)	Negative
14	72	M	BM	MPN with small monoclonal B-cell population	c.730A>C; p.T244P	3%	6	*JAK2* p.V617F (77%); *SRSF2* p.P95H (43%) *TET2* p.P739Lfs*12 (45%); *TET2* p.Q810* (46%)	Negative

Abbreviation: AML, acute myeloid leukemia; BM, bone marrow; ET, essential thrombocythemia; MDS, myelodysplastic syndrome; MPN, myeloproliferative neoplasm; N/A, not applicable; PB, peripheral blood.

## Data Availability

The data presented in this study are available on request from the corresponding author.

## Supplementary Materials

The following are available online at https://www.mdpi.com/article/10.3390/ijms25105183/s1, Table S1: List of genes analyzed in our study., Table S2: Pathological features in patients with myeloid neoplasm carrying *BRAF* mutations (n = 14)., Table S3: Clinical outcomes in patients with myeloid neoplasms carrying *BRAF* mutations (n = 14).
